# Evaluation of the Antifibrotic and Functional Effects of Pirfenidone in an Experimental Frozen Shoulder Model

**DOI:** 10.1002/jor.70210

**Published:** 2026-04-20

**Authors:** Sezer Astan, Orhan Balta, Fikret Gevrek

**Affiliations:** ^1^ Department of Orthopaedics and Traumatology Tokat Gaziosmanpasa University Hospital Tokat Turkey; ^2^ Department of Histology and Embryology Tokat Gaziosmanpasa University Hospital Tokat Merkez Tokat

**Keywords:** adhesive capsulitis, antifibrotic agent, dexamethasone, frozen shoulder, pirfenidone, rat model

## Abstract

This study aimed to investigate the histopathological and functional effects of pirfenidone in a rat model of frozen shoulder (adhesive capsulitis). Thirty‐two male Wistar Albino rats were randomly divided into four groups (*n* = 8 each): control, frozen shoulder (FS), pirfenidone‐treated (FS + PFD), and dexamethasone‐treated (FS + DEX). The FS model was induced by immobilizing the shoulder joint with plaster for 4 weeks. Treatment groups received oral pirfenidone (30 mg/kg/day) or prednisolone (3 mg/kg/day) for 30 days. Histopathological changes in the synovial membrane and fibrous capsule were evaluated with hematoxylin–eosin and Masson's trichrome staining, while shoulder joint range of motion (ROM) was measured. The FS group showed significant pathological alterations, including synovial epithelial atrophy, loss of synovial folds, hyperemia, edema, and severe capsular fibrosis. Both pirfenidone and dexamethasone treatments reduced these abnormalities. Pirfenidone was more effective in preserving collagen fiber organization and synovial fold integrity. ROM was markedly reduced in the FS group, but partial recovery occurred in both treatment groups. Pirfenidone provided greater functional improvement compared with dexamethasone. Inflammation scores did not differ significantly between groups, consistent with evaluation during the fibrotic phase. Pirfenidone attenuates fibrotic changes and improves joint mobility in an experimental frozen shoulder model. Pirfenidone improved histological fibrosis scores and ROM in this experimental model. Comparative effectiveness versus corticosteroids requires confirmation in larger, appropriately powered studies. These results emphasize the antifibrotic potential of pirfenidone and support further long‐term and clinical studies to clarify its role in adhesive capsulitis management.

## 1 Introduction

Frozen shoulder (adhesive capsulitis) is a common shoulder disorder characterized by progressive pain, capsular contracture, and limitation of joint motion [1, 2]. These symptoms, together with the often unfavorable prognosis, significantly impair physical health and quality of life. However, the exact pathophysiological mechanisms underlying frozen shoulder remain unclear, and there is still no consensus regarding the most effective treatment approach [1, 2]. Clinically, conservative treatment options such as analgesics, physical therapy, and corticosteroid administration are widely utilized [3]. Oral corticosteroids and intra‐articular corticosteroid injections can provide short‐term relief of pain and improvement in range of motion (ROM) by suppressing inflammation [3]; however, their long‐term efficacy is limited, and the risk of adverse effects remains considerable [4]. Moreover, these therapies often fail to halt the progression of fibrotic processes. Therefore, the development of novel, safe, and effective treatment alternatives is urgently needed.

Experimental models of frozen shoulder in rats have been shown to effectively mimic key pathological features of the human condition, including inflammation, fibrosis, and restricted joint mobility [5]. Among these, external immobilization is the simplest and most commonly employed method, whereas injection‐ and embolization‐based models allow for a more detailed investigation of molecular and vascular mechanisms underlying the disease [1, 2, 5, 6, 7]. Such models are indispensable for advancing the understanding of frozen shoulder pathogenesis and for preclinical evaluation of novel therapeutic approaches [5].

Pirfenidone is a well‐established antifibrotic agent primarily approved for the treatment of idiopathic pulmonary fibrosis, and it is currently under investigation for various other fibrotic conditions [8, 9]. Previous studies have demonstrated that pirfenidone can delay the progression of osteoarthritis by reducing synovial fibrosis and inflammation [9]. Due to its combined antifibrotic and anti‐inflammatory properties, pirfenidone has the potential to mitigate fibrosis in multiple organs and tissues. Although pirfenidone targets the transforming growth factor‐β (TGF‐β) signaling pathway—a key mediator in the pathogenesis of frozen shoulder—there are currently no peer‐reviewed studies evaluating its efficacy in animal models of shoulder fibrosis [1]. Evidence from hepatic and pulmonary fibrosis models indicates that pirfenidone exerts potent antifibrotic effects by interfering with platelet‐derived growth factor (PDGF) signaling and inhibiting fibroblast proliferation as well as extracellular matrix deposition via the TGF‐β1/Smad2/3 pathway [10, 11].

Given the widespread clinical use of oral corticosteroids for short‐term inflammation control in frozen shoulder, a direct comparison with pirfenidone—particularly in terms of long‐term antifibrotic and functional benefits—has become a scientific necessity. Such comparative studies may provide insights that move beyond symptomatic relief toward therapeutic strategies targeting the underlying fibrotic pathology of adhesive capsulitis. Therefore, the aim of this study was to evaluate the effects of pirfenidone on histological changes in the synovial membrane and fibrous capsule, as well as on joint ROM, in a rat model of glenohumeral joint immobilization. We hypothesized that pirfenidone would exert beneficial effects on the glenohumeral joint in a rat model of shoulder contracture induced by immobilization.

### 1.1 Materials and Methods

A total of 32 male Wistar Albino (WA) rats (7 weeks old, weighing 250–280 g) were housed under standard laboratory conditions (room temperature [RT] 20°C–24°C, relative humidity 40%–60%, and a 12‐h light/dark cycle). All animals were maintained in accordance with institutional guidelines and the National Research Council's Guide for the Care and Use of Laboratory Animals. The experimental protocol was approved by the Tokat Gaziosmanpaşa University Animal Experiments Local Ethics Committee (Approval No: 2024 HAYDEK‐15).

The rats were randomly divided into four groups, each consisting of eight animals: the Control group (C), the frozen shoulder model group (FS), the frozen shoulder + pirfenidone treatment group (FS + PFD), and the frozen shoulder + dexamethasone treatment group (FS + DEX).

**Figure 1:**
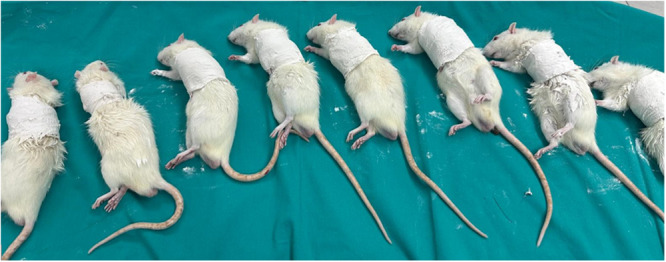
Immobilization is accomplished with applying molding plaster around the left whole arm.

Eight rats were housed per cage. After a 7‐day acclimatization period, a frozen shoulder model was induced in accordance with previously published protocols [5] by immobilizing the left shoulder joint with plaster for 4 weeks. The left shoulders of rats in the FS, FS + PFD, and FS + DEX groups were immobilized (Figure 1).

### 1.2 Statistical Analysis

Quantitative data were obtained regarding the arithmetic mean and standard deviation. Shapiro–Wilk's test was used, was examined to assess the data normality. One‐way analysis of variance test was used to compare all variables among groups. For post‐hoc comparisons between the pair‐wise groups, the Tukey HSD test was used. A *p*‐value < 0.05 was considered significant. Analyses were performed using SPSS 27 (IBM SPSS Statistics for Windows, Version 27.0. Armonk, NY: IBM Corp). For the ROM variation considered as the primer, it was found to work with a total of 28 rats, 7 rats in each group with 80% power, 5% type 1 error and an effect size of 0.7 by using G* power 3.1.9.6 program.

#### 1.2.1 Drug Administration

In the FS + PFD group, pirfenidone (Pirfa 200 mg, ARİS) was administered orally via gavage at a dose of 30 mg/kg/day for 30 consecutive days [12]. In the FS + DEX group, rats received an oral prednisolone solution (Deltacortil 5 mg, Pfizer) at a dose of 3 mg/kg/day by intragastric administration at 7:00 a.m. for 30 days. The prednisolone dosage was selected based on previous studies to achieve adequate anti‐inflammatory effects [12, 13]. Pirfenidone/prednisolone was initiated during the immobilization period (with plaster in place) on Day 30 and continued for 30 days. At the end of the 4‐week immobilization period, the plaster was removed in all immobilized groups.

#### 1.2.2 Anesthesia and Surgical Procedure

All rats were anesthetized via intraperitoneal injection of xylazine hydrochloride (Rompun 2%, Bayer, Turkey; 10 mg/kg) combined with ketamine hydrochloride (Alfamine 10%; 50–60 mg/kg). The left forelimbs of rats in the FS, FS + PFD, and FS + DEX groups were immobilized with plaster to maintain the shoulder joint in 90° internal rotation for 30 days, following the protocol described by Kim et al. [5].

## 2 Measurement of Shoulder Abduction Angle

**Figure 2:**
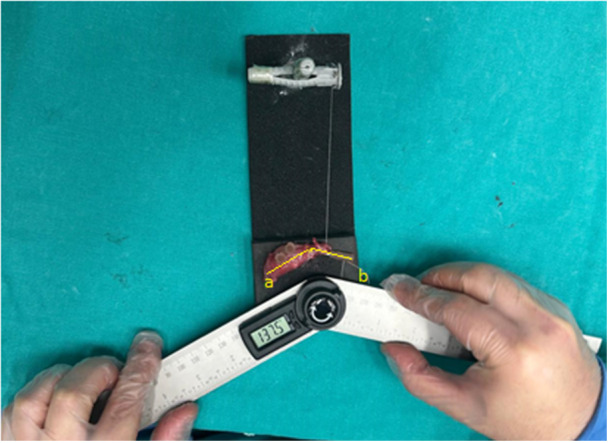
Measurement of the glenohumeral joint abduction angle (range of motion degrees). (Reference lines a: scapular spine, b: humeral axis).

Following sacrifice by cervical dislocation, the inferior border of the scapula was exposed, and the scapula together with the proximal two‐thirds of the humerus was removed en bloc. Measurements were performed before the shoulder joints began to degenerate. During the procedure, tissues were kept moist with sterile saline solution. For angle assessment, a syringe needle was inserted longitudinally into the humeral shaft, while another needle placed parallel to the scapular spine served as a reference point [14]. The shoulder joints were then positioned in maximum passive abduction by applying a constant torque of 10 g (3.92 × 10^−3^ N ∙ m) to the humeral shaft. Subsequently, the abduction angle was measured as the angle between the inferior border of the scapula and the humeral shaft (Figure 2) [15].

### 2.1 Histological Procedures

Following the experiment, the rat's shoulders were excised and fixed in 4% buffered neutral formalin solution (pH 7.2) for 48 h. The specimens were subsequently decalcified in a commercial decalcification solution. After fixation, tissues were washed under running tap water, dehydrated in graded ethanol series (70%, 80%, 90%, 96%, and 100%; 5 min each), cleared in xylene, and infiltrated in three successive paraffin baths at 60°C. The tissues were then embedded in fresh paraffin with identical orientation to ensure consistency, and serial sagittal sections of 4–5 µm thickness were obtained using a rotary microtome (Leica RM2135). The tissue sections were mounted on slides and stained with hematoxylin–eosin (H&E) and Masson's trichrome.

All histological evaluations were performed in a blinded manner by an experienced histologist.

### 2.2 Hematoxylin–Eosin (H&E) Staining

Thin tissue sections of the shoulder specimens, previously fixed in formalin and embedded in paraffin blocks, were subjected to deparaffinization and rehydration procedures. Sections were then stained in hematoxylin solution for 10 min, rinsed in running tap water for 5 min, briefly dipped in acid alcohol, and washed again under running water. They were subsequently immersed in eosin solution for 3 min, followed by several rinses in distilled water to remove excess stain.

The sections were then dehydrated through a graded ethanol series (80%, 90%, 95%, and absolute alcohol), cleared in xylene (3 changes × 15 min), and mounted with coverslips using Entellan. Prepared H&E stained slides were examined histologically under a light microscope (Nikon Eclipse 200; Nikon).

### 2.3 Masson's Trichrome Staining

Tissue sections of 4–5 µm thickness were first incubated at 60°C to melt the paraffin and subsequently deparaffinized in a series of xylene baths. After rehydration through descending alcohol series (from 100% to lower concentrations) to distilled water, the sections were immersed in Weigert's hematoxylin solution for 10 min. They were then rinsed in running tap water for 5 min, transferred to distilled water, and stained in acid fuchsin solution for 1 min. Following two washes in distilled water, the sections were treated with phosphotungstic acid solution for 10–15 min.

Afterwards, the sections were washed twice in distilled water and stained with aniline blue solution for 1 min, followed by another rinse in distilled water. The samples were then dehydrated through graded ethanol concentrations (80%, 90%, 96%, and 100%) and cleared in three successive xylene baths. Finally, the sections were mounted with coverslips using Entellan, resulting in permanent preparations suitable for microscopic analysis.

### 2.4 Histopathological Analyses

Histopathological evaluations were performed on an average of 7–8 consecutive sections from each specimen, stained with H&E and Masson's trichrome. Using these staining methods, the histological architecture of the glenohumeral joints was examined in detail. For histological assessment, the synovial membrane of the joint capsule and the surrounding collagen‐rich fibrous layers were analyzed under a light microscope.

Particular attention was given to Masson's trichrome‐stained sections, where histopathological changes were evaluated based on the criteria outlined in Table 1. Parameters included hyperemia, edema, epithelial atrophy, folding of the synovial membrane, and subsynovial fibrosis, as well as hyperemia, edema, angiogenesis, and capsular fibrosis within the fibrous capsule.

**Table 1:** Scoring criteria of histopathological changes in the glenohumeral joint synovial membrane and fibrous capsule.

		Synovial membrane	Fibrous capsule
Structural changes	Score	Criteria	Criteria
None	0	None	None
Weak	1	Hyperemia Mild synovial epithelium atrophy Minimally flattened synovial folds (5%–30%)	Hyperemia Angiogenesis (3–7 vessels/visual field (10×)) Mild capsular fibrosis (10%–20%)
Moderate	2	Edema Hyperemia Moderate synovial epithelium atrophy Partly flattened synovial folds (40%–60%) Mild‐moderate subsynovium fibrosis (20%–60%)	Edema Hyperemia Angiogenesis (8–12 vessels/visual field (10×)) Moderate capsular fibrosis (30%–60%)
Strong	3	Edema Hyperemia Severe synovial epithelium atrophy Mostly flattened synovial folds (70%–100%) Severe subsynovium fibrosis (70%–100%)	Edema Hyperemia Angiogenesis (> 12 vessels per visual field (10×)) Severe capsular fibrosis (70%–100%)
Inflammatory response			
None	0	None	None
Weak	1	Scattered/focal appearing inflammatory cells	Scattered/focal appearing inflammatory cells
Moderate	2	Several/multifocal appearing inflammatory cells	Several/multifocal appearing inflammatory cells
Strong	3	Large number of/multifocal confluent inflammatory cells	Large number of/multifocal confluent inflammatory cells

The inflammatory response was assessed semiquantitatively in H&E‐stained sections under light microscopy (Nikon Eclipse 200; Nikon), using the inflammation criteria also presented in Table 1 [16, 17, 18, 19, 20, 21, 22, 23, 24]. Semiquantitative scores obtained for each parameter were statistically compared as group mean values.

## 3 Results

End‐Point Assessments (Histology and ROM) Were Performed at Sacrifice After Completion of the 30‐Day Treatment Period.

One rat from each group was lost during follow‐up.

### 3.1 Histopathological Findings

**Figure 3:**
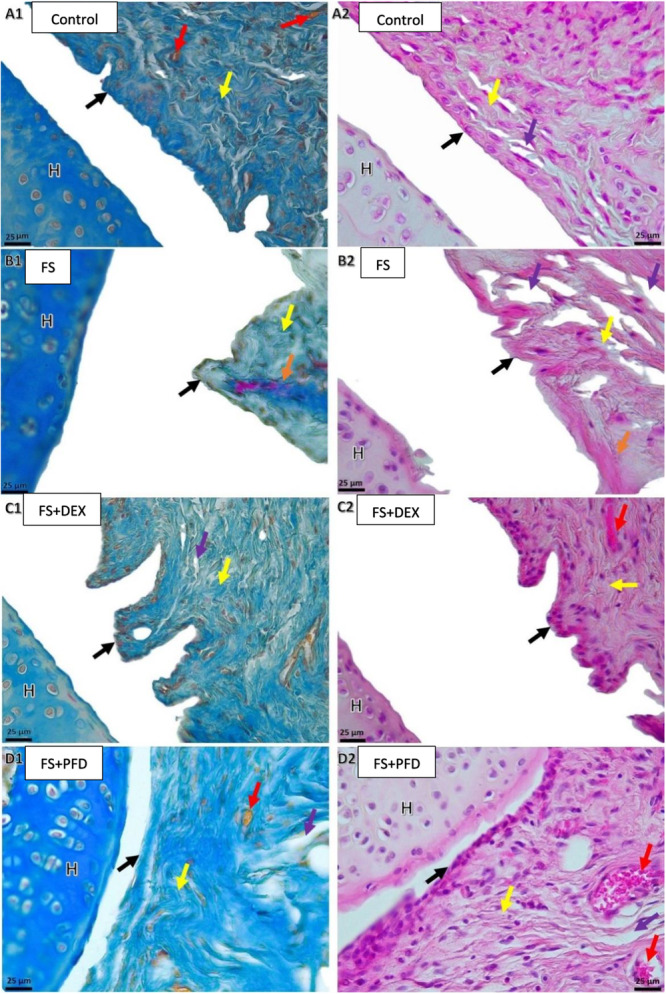
Representative microscopic images of the glenohumeral joint region from the study groups stained with Masson's trichrome (first column, left to right) and hematoxylin–eosin (second column, left to right). A1 and A2: Control; B1 and B2: Frozen shoulder; C1 and C2: Frozen shoulder + Dexamethasone; D1 and D2: Frozen shoulder + Pirfenidone group (Scale bars: 25 µm). H: humeral head. Black arrow: synovial epithelium; Yellow arrow: collagen fiber bundles; Red arrow: hyperemia and angiogenesis; Purple arrow: edema; Orange arrow: fibrosis.

Based on H&E and Masson's trichrome staining, the prominent histopathological observations were as follows:

In the control group, the synovial membrane displayed a normal epithelial structure, surrounded by regularly organized bundles of collagen fibers. The synovial capsule maintained its normal histological architecture without evidence of pathological changes (Figure 3, A1 and A2).

In the frozen shoulder model group, marked morphological disruption and structural irregularities were detected in the capsular tissue. These alterations included synovial epithelial atrophy, flattening of the synovial folds, hyperemia and edema in the surrounding synovial capsule, capsular fibrosis, and disorganization of collagen fiber bundles (Figure 3, B1 and B2).

In the treatment groups (dexamethasone and pirfenidone), improvement in the histopathological changes observed in the frozen shoulder model was evident. In the dexamethasone‐treated group, preservation of a greater number of synovial cells and epithelial folds was noted compared with the pirfenidone group. Areas of hyperemia and edema appeared less prominent. In both treatment groups—particularly in the dexamethasone group—the collagen fiber bundles of the synovial capsule demonstrated a more regular organization, resembling that of the control group (Figure 3, C1, C2, and D1, D2).

Microscopic analysis of H&E‐stained glenohumeral sections revealed no significant differences in inflammatory response scores among the groups.

Significant differences were observed among the groups in terms of synovial membrane structural changes (SMSC), fibrous capsule structural changes (FCSC), and ROM (Table 2).

**Table 2:** Mean histopathological analysis scores of glenohumeral joint tissues by group.

Variables	Total	Groups				*p*
		Control (*n* = 7)	Frozen shoulder (*n* = 7)	Frozen shoulder + pirferidone (*n* = 7)	Frozen shoulder + steroid (*n* = 7)	
SMSC	0.92 ± 0.69	0.18 ± 0.06 (a)	1.98 ± 0.26 (b)	0.67 ± 0.1 (c)	0.86 ± 0.2 (c)	< 0.001
FCSC	0.96 ± 0.76	0.21 ± 0.07 (a)	2.11 ± 0.26 (b)	0.71 ± 0.2 (c)	0.81 ± 0.38 (c)	< 0.001
ROM	127.86 ± 14.3	148.71 ± 3.99 (a)	111.29 ± 2.56 (b)	129.71 ± 2.14 (c)	121.71 ± 4.19 (d)	< 0.001

*Note:* Distribution of variables across groups. Data are shown as Mean ± Standard Deviation. p: One‐Way ANOVA was used. (abcd): In same row, Common letters indicate statistical insignificance.

Abbreviations: FCSC, fibrous capsule structural changes; ROM, Glenohumeral ROM across groups of rats; SMSC, synovial membrane structural changes.

### 3.2 Synovial Membrane Structural Changes (SMSC)

**Figure 4:**
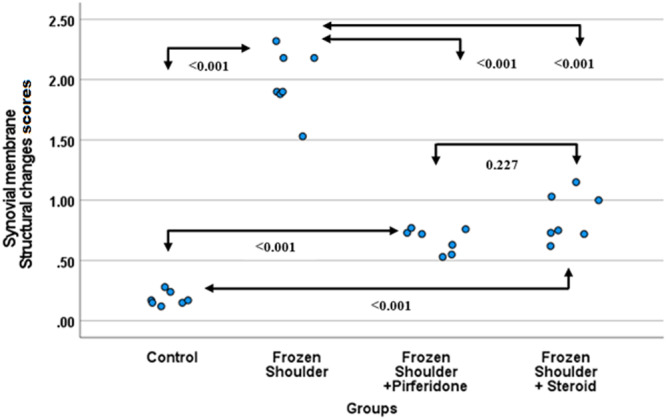
Beeswarm plot graph of SMSC by groups. SMSC, synovial membrane structural changes.

The lowest SMSC values were recorded in the control group (0.18 ± 0.06). In the frozen shoulder group (1.98 ± 0.26), these values increased significantly and were statistically different from all other groups (*p* < 0.001). In the pirfenidone (0.67 ± 0.1) and steroid (0.86 ± 0.2) treatment groups, the scores remained at intermediate levels and were comparable to each other. These findings indicate that structural changes in the synovial membrane increased markedly in the frozen shoulder model, whereas both treatment approaches significantly attenuated this pathological process (Figure 4).

### 3.3 Fibrous Capsule Structural Changes (FCSC)

**Figure 5:**
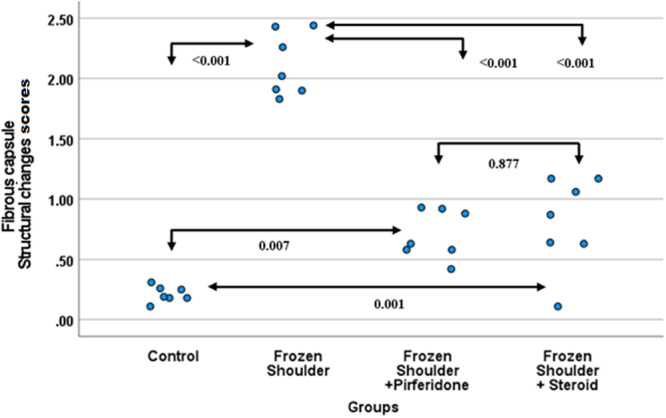
Beeswarm plot graph of FCSC by groups. FCSC, fibrous capsule structural changes.

The lowest FCSC values were detected in the control group (0.21 ± 0.07), whereas the frozen shoulder group (2.11 ± 0.26) demonstrated the highest degree of fibrosis (*p* < 0.001). In the pirfenidone (0.71 ± 0.2) and steroid (0.81 ± 0.38) treatment groups, values were similar to each other, with no statistically significant difference observed between them. These findings indicate that capsular fibrosis was markedly increased in the frozen shoulder model, but both pirfenidone and steroid treatments significantly reduced this pathological progression (Figure 5).

### 3.4 Range of Motion (ROM)

**Figure 6:**
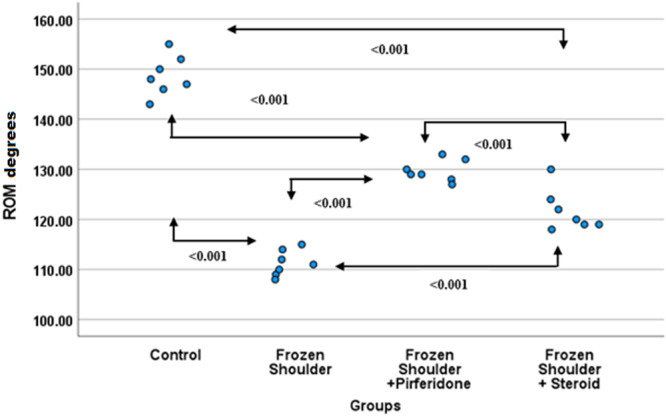
Beeswarm plot graph of ROM by groups. ROM, range of motion.

The highest ROM values were observed in the control group (148.71 ± 3.99). In the frozen shoulder group, ROM was significantly reduced (111.29 ± 2.56), and this decrease was statistically different from all other groups (*p* < 0.001). When comparing the treatment groups, ROM values were better preserved in the pirfenidone group (129.71 ± 2.14) compared with the steroid group (121.71 ± 4.19). ROM values were numerically higher in the pirfenidone group compared with the steroid group (Figure 6).

## 4 Discussion

In this experimental study, the histopathological and functional effects of pirfenidone treatment were evaluated in a plaster‐induced frozen shoulder (adhesive capsulitis) model. The findings demonstrated that the antifibrotic properties of pirfenidone led to significant improvements both in structural tissue alterations and in joint ROM.

Frozen shoulder is a complex clinical condition characterized by progressive pain and limitation of motion, beginning with synovial inflammation and advancing to capsular fibrosis. The 4‐week immobilization model used in our study reliably simulated the fibrotic phase and reproduced histopathological features consistent with those described in the literature.

Gu et al. reported in their study on Sprague–Dawley rats that immobilization by plaster for 1, 2, 3, and 4 weeks resulted in distinct histological changes: synovial and capillary proliferation during Weeks 2–3, and subscapular adhesions with type I and III collagen accumulation during Weeks 3–4 [2]. Similarly, according to Kim et al., inflammation predominates in the first 2 weeks, while fibrosis becomes dominant by Week 3, at which point the model progresses to the “frozen” stage. They summarized their findings as: *“This model was attained within 3 weeks after immobilization”* [5]. In line with this, Ahn et al. also described the freezing phase as corresponding to 3 days of immobilization (inflammation) and the frozen phase as corresponding to 3 weeks of immobilization (fibrosis) in rats [25].

Previous studies have shown that the TGF‐β1/Smad signaling pathway plays a central role in the pathogenesis of frozen shoulder [1]. The 4‐week plaster immobilization model applied in this study successfully reproduced the pathological features of the “frozen phase” described in the literatüre [2, 5]. The histopathological findings observed in our model—including synovial epithelial atrophy, loss of synovial folds, and disruption of collagen fiber organization—further confirm that this model accurately reflects the late fibrotic stage.

In the pirfenidone‐treated group, structural damage in both the synovial membrane and the fibrous capsule was significantly reduced. Moreover, a marked improvement in joint ROM was observed. Given pirfenidone's known antifibrotic actions in other tissues, the observed reduction in capsular fibrosis is consistent with reduced fibroblast‐driven matrix deposition; however, direct assessment of fibroblast proliferation/activation markers is required to confirm this mechanism in our model [2, 10, 11]. To our knowledge, this is the first study to demonstrate similar effects in a frozen shoulder model.

Histologically, preservation of collagen fiber organization and maintenance of synovial fold integrity were consistent with the functional improvements observed. Because assessments were performed at a single endpoint, we cannot fully exclude time‐dependent spontaneous remodeling. However, the untreated FS group retained pronounced fibrotic changes at the same timepoint, supporting a treatment‐associated improvement. Interestingly, in some pirfenidone‐treated specimens, the synovium appeared more apposed to the humeral head; whether this reflects sectioning variability or treatment‐related synovial remodeling requires dedicated investigation. Future studies incorporating serial timepoints would better delineate the natural course versus treatment effects. These findings suggest that pirfenidone is associated with attenuation of fibrotic tissue remodeling and preservation of joint function in this experimental model. The substantial histological and functional improvements observed with pirfenidone treatment are consistent with an antifibrotic effect characterized by reduced extracellular matrix accumulation and improved tissue organization. However, direct assessment of fibroblast activation or proliferation was not performed in this study, and therefore mechanistic conclusions should be interpreted with caution.

Although steroid administration also contributed to histopathological improvement, the ROM values remained lower than those observed in the pirfenidone group. This finding suggests that while corticosteroids are effective in suppressing inflammation, they may be insufficient in halting the fibrotic process. Indeed, previous studies have similarly reported that corticosteroids provide marked short‐term benefits but have limited long‐term efficacy [3, 4]. This supports the notion that corticosteroids effectively reduce the acute inflammatory response but fail to prevent fibrosis progression. No significant differences in inflammation scores were observed among the groups, likely because evaluations were conducted during the fibrotic phase of the model, when inflammatory activity becomes secondary to capsular fibrosis. Accordingly, the observed effects of pirfenidone are more consistent with antifibrotic tissue remodeling rather than anti‐inflammatory activity. Although inhibition of TGF‐β1/Smad2/3 and PDGF signaling has been reported in other fibrotic disease models, these pathways were not directly assessed in the present study and therefore remain hypothetical in this context. [9, 12].

The strengths of our study include its randomized group design, blinded histological evaluation, and the combined assessment of both morphological and functional parameters. However, certain limitations should be acknowledged. The treatment duration was limited to 30 days, and thus, long‐term effects were not assessed. A dose–response relationship for pirfenidone was not investigated. Moreover, molecular markers such as TGF‐β1 or Smad protein expression were not analyzed. Addressing these limitations in future studies will allow for a more comprehensive understanding of the therapeutic potential of pirfenidone. A key limitation of this study is the lack of cellular and molecular analyses (e.g., Ki67/PCNA, α‐SMA, COL1A1/COL3A1, and direct assessment of TGF‐β1/Smad2/3 or PDGF signaling), which precludes definitive mechanistic conclusions; future studies integrating these endpoints and in vitro assays are warranted.

## Author Contributions

**Sezer Astan:** concept, design, data analysis, manuscript writing. **Orhan Balta:** data collection, critical revision. **Fikret Gevrek:** histological analysis, interpretation.

## Conflicts of Interest

The authors declare no conflicts of interest.
